# Effects of invisible orthodontic retainers on masticatory muscles activity during sleep: a controlled trial

**DOI:** 10.1186/s40510-018-0228-y

**Published:** 2018-07-23

**Authors:** Daniele Manfredini, Luca Lombardo, Letizia Vigiani, Angela Arreghini, Giuseppe Siciliani

**Affiliations:** 1 Postgraduate School in Orthodontics, Ferrara University, Ferrara, Italy; 2 School of Dentistry, University of Siena, Siena, Italy

**Keywords:** Sleep bruxism, Masticatory muscles activity, Orthodontics, Retainers

## Abstract

**Background:**

This study aims to evaluate if invisible orthodontic retainers can affect sleep-time masticatory muscle activity (sMMA) over a short-term period in healthy individuals.

**Methods:**

Nineteen (*N* = 19) healthy subjects underwent an in-home evaluation with a portable device for electromyographic (EMG) assessment. The study protocol provided two baseline recording nights, a night off, and then two additional nights with passive customized orthodontic retainers in situ. For each recording night, the sleep bruxism (SB) index (i.e., average number of SB events/hour) and the overall number of masseter muscle contractions were assessed. Comparison between values gathered over the four recording nights was made with a parametric test, based on the null hypothesis that there was no difference between wearing or not wearing the retainers as far as the sMMA variables are concerned.

**Results:**

Average SB index of the first two nights without the retainers was 3.0 ± 1.5, whilst the average values with the retainers in situ was 3.6 ± 1.9. ANOVA test showed the absence of significant differences between the four nights. Similarly, no differences were shown between the four nights as for the total number of sMMA events. Based on that, the null hypothesis was not rejected.

**Conclusions:**

Findings suggest the absence of relevant effects of invisible orthodontic retainers on sMMA in healthy individuals during the short-term period.

## Background

The effects of oral appliances (OA) on sleep-time masticatory muscles activity (sMMA) have been much debated over the past few decades [1]. In particular, among the proposed mechanisms of action to explain their effectiveness on temporomandibular disorders (TMD) symptoms, it has been suggested a decrease in the electromyographic (EMG) activity of jaw muscles [2]. Actually, such hypothesis has never been convincingly confirmed and is not fully supported by the literature. For instance, a recent review on bruxism management pointed out that the effects of OA on jaw muscles activity, as measured with polysomnography (PSG) or home EMG recordings, are not unequivocal [3].

Within this complex field, things are further complicated by the emerging alert concerning the possible relationship between the use of orthodontic aligners and invisible retention devices and the onset of pain in the jaw muscles, which has been occasionally described [4]. This suggests that the effects of such appliances on masticatory muscles activity should be assessed. In particular, concerns are raised based on past data suggesting that soft appliances may even increase the jaw muscles EMG activity during sleep time with respect to baseline values as well as to hard-resin appliances [5].

Invisible orthodontic devices are not comparable to OA that are commonly used in TMD patients, because they are neither rigid as a conventional appliance nor soft as over-the-counter devices [6]. At present, there are no data on their effects on sMMA parameters. For instance, as for the aligners, there are contrasting opinion-based claims on the fact that they could increase or decrease TMD symptoms and/or EMG activity [7]. A basic strategy to get deeper into the issue should be to gather data on passive invisible devices first (i.e., retainers).

Whilst there are objective technical difficulties to perform studies adopting the reference approach to assess jaw muscles activity that is specifically associated with sleep bruxism (SB), viz., PSG/SB [8], recent advances in the design and diffusion of reliable devices for in-home measurement of jaw muscles’ EMG activity during sleep have provided valid alternative for a user-friendly approach to sMMA assessment [9, 10].

Based on these premises, this investigation assessed sMMA in healthy individuals by using a portable home-EMG/ECG recorder. The study had an alternate design, with all subjects undergoing both a two-night recording without and another two-night with an invisible orthodontic retainer in situ. The null hypothesis was that no differences existed between wearing and not wearing aligners in the short-term period as far as sMMA parameters are concerned.

## Methods

The needed sample size was assessed based on the minimum requirement of a 5% alpha error and 80% statistical power [11]. The difference to detect (effect size) was set equal to the variance of the main outcome variable (i.e., number of MMA events per sleep hour). Based on those calculations, a study sample of 19 healthy volunteers (14 females; mean age 28.3 ± 2.4, range 25–35 years) was recruited among the staff of the Post-Graduate School in Orthodontics, University of Ferrara, Ferrara, Italy.

Potential participants were excluded based on the presence of any painful temporomandibular disorders, as screened with the Diagnostic Criteria for Temporomandibular Disorders (DC/TMD) guidelines [12], and/or a documented history of neurological, psychiatric, or sleep disorders. All individuals were asked to avoid assuming potential sleep-altering drugs or substances (e.g., nicotine, alcohol, caffeine) within the hours that preceded recordings.

All subjects underwent an in-home evaluation with a portable device (Bruxoff®, OT Bioelettronica, Torino, Italy) that provides a simultaneous recording of EMG signals from both the masseter muscles as well as hearth frequency. The study design provided two consecutive recording nights, a night off, and then two additional recording nights with orthodontic retainers in situ. Technical details about the device as well as the recording procedure have been described elsewhere [13, 14]. Previous studies showed that the device allows a good repeatability of findings [13] as well as high sensitivity (92.3%) and specificity (91.6%) for SB diagnosis when the diagnostic cut-off was set at 4 SB episodes per hour [14], as suggested by PSG/SB criteria [15, 16].

In this investigation, both the SB events (i.e., masseter contractions exceeding the 10% of maximum voluntary contraction [MVC] amplitude and preceded by a 20% increase in hearth rate) and the overthreshold masseter contractions not preceded by the hearth rate increase were recorded. The latter were here called sMMA events. For each recording night, the number of SB episodes per sleep hour (SB index), the number of phasic, tonic, and mixed sMMA events per hour as well as the total sMMA events per night were calculated.

For each individual, two orthodontic retainers, made of the same material that is commonly used to build invisible aligners, were manufactured. The retainers, one for the upper and one for the lower arch, were built on digital casts, as created after acquiring digital images of dental arches with an intraoral scanner (Trios 3 Shape®, 3 Shape A/S, Copenhagen, Denmark). The same investigator with a 3-year post-graduate education in orthodontics (A.A.) manufactured the retainers for all participants by using a 1-mm-thick thermoplastic resin foil (Duran®, Sheu Dental, Iserlhorn, Germany). The appliances were designed to cover all teeth up to the second molars. They were passively adapted to each tooth, to avoid any discomfort during the recording nights, and participants were asked to check for passive fit before starting any recordings.

The procedures were approved by the Internal Review Board (IRB) of the Post-Graduate School of Orthodontics, University of Ferrara, Ferrara, Italy (Prot. #032_2016). All individuals gave their informed consent in accordance with the Helsinki Declaration and understood that they were free to withdraw from the experiment at any time.

For all variables, descriptive statistics (i.e., average values/night) were calculated, and Kolmogorov-Smirnov test was used to assess normality of distribution. Analysis of variance (ANOVA) with Bonferroni’s post-hoc analysis, when needed, was used to test for significant differences between average values of the four recording nights in all the outcome variables. Statistical significance was set at *p* < 0.05. All procedures were performed with the Statistical Package for Social Sciences (SPSS 21.0, IBM, Milan, Italy).

## Results

Demographic data of the study population are presented in Table 1.

**Table 1:** Demographic data of the study population

No. of participants	Females/males	Mean age (s.d.)	Age range
19	14/5	28.3 (2.4)	25–35

*s.d*. standard deviation

Mean sleep duration was 6.4 ± 1.6 h during the nights without and 6.6 ± 1.5 during the nights with the retainers (*p* > 0.05), thus suggesting that wearing or not wearing the retainers made no difference in terms of sleep duration in the short-term period. No relevant sleep interruptions that might have influenced results have been reported by any participants.

During the two nights without the retainers, five subjects had a SB index higher than the suggested threshold for PSG/SB diagnosis (i.e., > 4). Two additional subjects scored an average index higher than 4 during the nights with the retainers.

Average SB index of the study population over the four recording nights was 3.3 ± 1.7 (range 0.2–7.9), with mean values of 3.0 ± 1.5 during the two nights without and 3.6 ± 1.9 with the retainers. As for sMMA, average number of events/night was 73.4 ± 61.2. The mean number of phasic and tonic events/hour was 7.4 ± 5.0 and 5.0 ± 1.4, respectively (Table 2). The distribution of contraction types was similar between the four recording nights (Table 3).

**Table 2:** Average values (standard deviation (s.d.)) of outcome variables during the four recording nights

Outcome variable	Average values (s.d.)	Range
SB index	3.3 (1.7)	0.2–7.9
Total number of sMMA events	73.4 (61.3)	6–438.1
Phasic sMMA events per hour	7.4 (5.0)	0–20
Tonic sMMA events per hour	5.0 (4.6)	0–17
Mixed sMMA events per hour	1.4 (1.7)	0–6.0

**Table 3:** Percentage distribution of phasic, tonic, and mixed sMMA events over each recording night

Night #	Phasic sMMA events (%)	Tonic sMMA events (%)	Mixed sMMA events (%)
1	56.3	33.9	9.8
2	59.1	31.4	9.5
3	51.7	35.8	12.5
4	47.9	41.6	10.5

The average number of total sMMA events during the two nights without the retainers was 78.6 ± 65.6, and it was 67.9 ± 47.6 during the nights with the retainers. Between-night differences were not significant for any of the outcome variables (Table 4).

**Table 4:** Average values (standard deviation (s.d.)) of outcome variables over each recording night and ANOVA test for significant differences

Outcome variable	Night #	Average values (s.d.)	Range	ANOVA	Sig.
SB index	1	3.3 (1.6)	0.3–6.2	0.856	0.468
	2	2.8 (1.5)	0.2–5.9		
	3	3.5 (1.7)	1.2–6.5		
	4	3.7 (2.2)	0.6–7.9		
Total number of sMMA events	1	84.4 (94.0)	6–438	0.323	0.809
	2	72.9 (36.6)	23–153		
	3	71.5 (66.4)	21–311		
	4	64.9 (29.0)	11–130		
Phasic sMMA events/hour	1	8.2 (5.5)	1–20	0.864	0.464
	2	8.5 (4.9)	1–19		
	3	6.5 (4.2)	2–17		
	4	6.5 (5.3)	0–18		
Tonic sMMA events/hour	1	4.9 (3.9)	0–13	0.373	0.773
	2	4.5 (4.2)	1–14		
	3	4.5 (5.3)	0–17		
	4	5.9 (5.1)	0–17		
Mixed sMMA events/hour	1	1.4 (1.7)	0–6	0.190	0.903
	2	1.4 (1.8)	0–6		
	3	1.6 (1.7)	0–5		
	4	1.2 (1.7)	0–6		

## Discussion

The effects of oral appliances on sleep-time masticatory muscles activity have been studied from multiple perspectives over recent decades, viz., either as a possible device to decrease sleep bruxism, to reduce temporomandibular disorder pain, or even as a possible source of increased sMMA [1]. The most recent review on bruxism management found that the effects of OA treatment protocols on SB parameters are variable and hard to interpret [3].

Available literature suggests that, as far as the reduction of polysomnography (PSG)-assessed SB is concerned, the effects of occlusal devices are not unequivocal [17–21]. All papers are based on the use of hard-resin appliance and seem to indicate that a potential novelty effect is associated with a decrease in sleep-time EMG activity of jaw muscles, when occurring. On the other hand, soft appliances seem to have a less marked effect on the pattern of jaw muscles activity, as recorded during clenching on appliances tasks, with respect to hard-resin devices [22]. Such finding may explain their partial clinical ineffectiveness in patients with temporomandibular disorders [23]. In addition, they have been reported to potentially increase night-time EMG activity in about half individuals [5]. Possible clenching-inducing mechanism can be called into cause to explain these findings, which led to the progressively diminished use of soft and over-the-counter splints [6].

Within this complex field, the increasing diffusion of invisible orthodontic techniques has recently led to cautionary statements about their widespread use, based on their potential side effects [4, 24]. Among the various issues that must be clarified as far as the safety of invisible orthodontics is concerned, there is a need to study their effects on sleep-time MMA. Indeed, from a technical viewpoint, aligners cannot be compared to any available device that has been studied so far. They are neither hard nor soft, since they are built with a low-thickness hard thermoplastic resin foil and represent a hybrid solution as far as the resiliency of the material is concerned. Besides, the thermoplastic foil does not allow a control of the thickness with respect to usual procedures to build hard resin appliances. Thus, their effects on sMMA are likely comparable to neither hard- nor soft-resin appliances.

As a first step toward the study of the relationship between active aligners and sMMA, invisible orthodontic retainers were used in this investigation to test their short-term effects on masticatory muscles activity of healthy individuals. The absence of mechanical forces applied to the teeth was, in our intention, a needed criterion to start getting deeper into the issue by gathering a set of data for future comparisons with conditions in which orthodontic stimuli are applied to the teeth.

Participants to this study protocol wore a retainer both in the upper and lower arch, as per typical invisible orthodontic treatment. Such condition is not comparable to the available studies on the effects of OA on SB, which used a single appliance, usually in the maxillary arch. The fact of achieving an increase in the vertical dimension of occlusion by two appliances instead of one and having interarch appliance-to-appliance instead of teeth-to-appliance contacts are issues to consider when framing these findings within current knowledge on the topic.

Within these premises, an absence of differences in sMMA during the nights with and without the orthodontic retainers in situ was shown, thus not rejecting the null hypothesis that wearing or not wearing them makes no difference in the short-term period as for any sMMA parameters. Notwithstanding, it should be remarked that, despite the absence of differences between the nights with and without the retainers, a trend for a slight increase in sMMA with the retainers in situ has been observed. If confirmed, findings should be viewed as a cautionary remark toward the use of aligners in patients that are potentially at risk of developing pain in the jaw muscles. Screening individuals based on the presence of clinical signs of bruxism, viz., probable bruxism [8], seems a reasonable strategy to select individuals that can undergo safe invisible orthodontics.

Such finding may be worthy of further exploration by a possible refinement of the study design. To do that, a time control design with crossover design and random sequence of recordings as well as a longer recording period could help refining the study protocol. In addition, an involvement of general population individuals in future studies could help estimating the possible influence on results of a sample of subjects involved in the activity of dental clinics. Further recommendations concern the possible inclusion of a control group of individuals wearing active aligners, to really mimic the clinical conditions of an orthodontic treatment.

Along with considerations related with the study hypothesis, the data suggest a few observations for clinical research purposes. Indeed, average values for SB index over the four recording nights (i.e., 3.3 events/h) are only slightly lower than the cutoff threshold that is currently adopted to diagnose high frequency SB (i.e., 4 events/h). Based on such threshold, five subjects should be diagnosed as high-frequency sleep bruxers during the first two nights, and two additional individuals also scored higher than 4 events/h during the two nights with the retainers in situ. These findings, which have been gathered in a population of healthy asymptomatic volunteers, further support the need for a redefinition or elimination of a yes/no approach to SB assessment based on predefined thresholds for disease and for a better evaluation of the features of additive bruxism in selected populations [25, 26].

Besides, it should be pointed out that the amount of sMMA that is not classified as SB is quite relevant, with up to more than 70 events/night. Despite the absence of an audio-video recording provided a more balanced distribution between short- and longer-lasting events than reported in the literature considering only episodes with tooth grinding [27, 28], it is recommendable that future studies assess the relative importance of grinding and clenching episodes in terms of muscle work [29]. An evaluation of the duration of the activity is a needed strategy to pursue that goal, possibly thanks to a refinement of software that are currently used for an automatic analysis of EMG traces based on the raw number of events.

As a concluding general remark, it should be borne in mind that these findings should be appraised within the current need of assessing the emerging multifaceted clinical challenges that involve multispecialist evaluations in the dental profession. Orthodontists performing invisible orthodontic treatments can do it with a relative safety as for the risk of increasing sMMA, but this risk cannot be completely ruled out until more specific study protocols are performed to understand the outcomes at the individual level.

## Conclusions

This investigation assessed the short-term effects of invisible orthodontic retainers on sleep-time MMA in 19 healthy individuals by the use of a portable recorder in home environment. All individuals underwent a two-night recording without the retainers, then had a night off, and other two nights wearing the retainers. Findings showed that all sMMA parameters did not change significantly over the four recording nights.
